# Caminhada de Longa Duração pode Alterar Biomarcadores Cardíacos e Variáveis Ecocardiográficas Relacionadas à Função Diastólica?

**DOI:** 10.36660/abc.20200457

**Published:** 2020-10-13

**Affiliations:** 1 Universidade Federal do Rio Grande do Sul Programa de Pós-graduação em Ciências da Saúde: Cardiologia e Ciências Cardiovasculares Porto AlegreRS Brasil Programa de Pós-graduação em Ciências da Saúde: Cardiologia e Ciências Cardiovasculares - Universidade Federal do Rio Grande do Sul (UFRGS), Porto Alegre, RS – Brasil

**Keywords:** Caminhada, Esforço Físico, Biomarcadores, Pressão Sanguinea, Troponina T, Peptídeo Natriurético Encefálico, Creatinina Quinase, Ecocardiografia/métodos

A caminhada é uma das atividades físicas mais praticadas em todo o mundo. Também está inserida em programas de exercício físico com a finalidade de melhorar parâmetros relacionados à saúde em diferentes populações.^1^^,^^2^ Dessa maneira, cada vez mais aumenta a procura por eventos amadores que envolvam caminhada, principalmente aqueles conectados à natureza e à cultura de uma determinada região. Um dos eventos amadores mais conhecidos no mundo que envolve caminhada é o “Caminho de Santiago de Compostela”. Além disso, aqui no Brasil possuímos a “Rota das Missões”, localizada no Sul do país. Os trajetos podem ter distâncias variadas, podendo chegar em torno de 40km de caminhada em um mesmo dia. Esses são dois exemplos de atividades que envolvem caminhada e agregam uma experiência cultural ao evento.

Além dos dois eventos citados, existem as caminhadas ecológicas de longa distância, onde os participantes experimentam um contato direto com a natureza, passando por diferentes características de terrenos em um mesmo dia e podendo chegar a uma distância total percorrida de até 250 km em 4 dias de evento. Sabemos que cada indivíduo responde de maneira diferente a determinado estímulo, no entanto, percorrer mais de 50 km por dia com aclives, declives e terrenos acidentados, pode ter um impacto fisiológico negativo para qualquer indivíduo que se submete a esse tipo de modalidade.^3^ Além do dano fisiológico, nos preocupamos também com o impacto sobre o sistema cardiovascular em relação ao volume e a intensidade desse tipo de modalidade.^4^^,^^5^

Dentre os marcadores fisiológicos mais conhecidos, podemos citar alterações em biomarcadores cardíacos como a Creatina Quinase fração MB (CK-MB, *Creatine Kinase* MB), a Troponina T cardíaca (cTnT – *cardiac Troponin T*) e o aminoácido precursor N-terminal do peptídeo natriurético cerebral tipo B (NT-proBNP, *N-terminal pro B-type natriuretic peptide*), os quais podem estar relacionados com dano miocárdico.^4^ Dentre os marcadores de alteração da função cardíaca, podemos destacar algumas variáveis ecocardiográficas, tais como as velocidades diastólicas precoces (E) e tardia (A) transmitral, assim como a relação E/A e também avaliação tecidual da velocidade diastólica precoce (E'), todas podendo ser utilizadas para analisar disfunção diastólica.^6^

As evidências científicas disponíveis sobre os efeitos da caminhada de longa distância sobre biomarcadores cardíacos e função diastólica apresentam resultados divergentes em relação ao risco cardiovascular para esse tipo de modalidade. Nesta edição da revista, Euzébio et al.^7^ exploram essa questão em participantes de caminhada de longa distância (média de idade 46 ± 10,5). Os autores verificaram esses efeitos sobre o comportamento da função diastólica, por meio da onda E, A e relação E/A, além dos biomarcadores cardíacos CK-MB, Troponina T e NT-proBNP. Eles também avaliaram correlações entre as variáveis da função diastólica, entre os biomarcadores cardíacos e se existe correlação entre alguma variável da função diastólica com algum biomarcador cardíaco. Para isso, os autores conduziram um estudo longitudinal com 25 participantes adultos (todos homens), sendo que quatro mulheres foram excluídas por não preencherem o critério de seleção prévia, o qual consistia em percorrer 56 km, divididos em dois dias, em até três horas e dez minutos para os homens e três horas e 30 minutos para as mulheres. As avaliações foram divididas em cinco etapas, A0, momento anterior ao início do evento; A1, primeiro dia após término do trajeto; A2, segundo dia após término do trajeto; A3, terceiro dia após término do trajeto e A4, quarto dia após término do trajeto. O percurso total teve uma distância de 244,7 km. Como resultados principais, os autores encontraram aumentos significativos na CK-MB de A0 para A2, NT-proBNP de A0 para A1, A2, A3 e A4 e onda E' de A0 para A1. Curiosamente, e diferente de outros estudos, a Troponina T não apresentou diferenças significativas, assim como as ondas E e A. Além disso, foram identificadas correlações positivas entre CK-MB e NT-proBNP, CK-MB e Troponina T, entre E/A e E', e correlação negativa entre CK-MB e E/A.

De modo geral, o presente estudo nos traz resultados importantes sobre os efeitos da caminhada de longa distância sobre parâmetros relacionados à biomarcadores cardíacos e função diastólica, demonstrando que, apesar das alterações encontradas, não há critérios sugestivos de dano miocárdico, principalmente por não encontrarem alterações em Troponina T, onda E e onda A.^7^^,^^8^ Alterações encontradas para níveis séricos de NT-proBNP demonstram o efeito natriurético no mecanismo fisiológico de adaptação aguda e subaguda do sistema cardiovascular em relação ao esforço.^9^ Também é importante ressaltar que o aumento dos níveis séricos de CK-MB pode estar relacionado principalmente com temperaturas mais elevadas em determinados trechos (variou entre 19 e 32 ºC), e também com o tipo de terreno, distância percorrida e intensidade em cada trecho. Justamente a variação encontrada nessa variável foi entre A0 e A2, onde era predominantemente declive, o qual promove maior contração muscular excêntrica, e que está diretamente relacionada com maior dano muscular, o que pode explicar tal comportamento, além de existir um efeito cumulativo entre o trecho A1 e A2.^10^ Em relação a função diastólica, o aumento significativo apenas da onda E' entre A0 e A1 pode ser explicado pela população estudada. Sabemos que indivíduos com menor condicionamento físico ficam mais suscetíveis a alterações na função diastólica quando expostos ao esforço físico comparados a indivíduos com maior condicionamento físico. Além disso, o treinamento físico regular pode minimizar alterações agudas da função diastólica em relação a maiores demandas cardiovasculares durante exercício prolongado e intenso.^11^ Em resumo, precisamos ficar atentos principalmente ao comportamento da Troponina T, a qual é específica para identificar dano miocárdico por isquemia, demonstrando que os danos ao sistema cardiovascular parecem ser mínimos e que essa modalidade de exercício parece ser segura.^12^

Em relação às correlações encontradas, podemos destacar a associação negativa entre CK-MB com E/A (relação da velocidade de enchimento ventricular rápido com a velocidade de contração atrial). Uma das especulações em relação a essa associação, pode ser atribuída ao quadro reversível de disfunção diastólica como um dos principais mecanismos de aumento plasmático da CK-MB, com uma consequente redução da onda E e aumento da onda A. Entretanto, esse assunto ainda precisa ser melhor explorado no que tange as relações entre biomarcadores cardíacos e função diastólica na ótica da caminhada de longa distância.

O estudo possui algumas limitações, dentre elas podemos destacar a ausência de padronização em relação ao horário da coleta de dados durante os dias do evento, visto que isso pode interferir diretamente nas avaliações dos níveis agudos dos biomarcadores cardíacos. Também seria importante coletar as mesmas variáveis em repouso sempre no dia seguinte ao percurso. Entretanto, os autores ressaltam que isso era inviável devido ao horário de início dos trajetos (4h da manhã). Aqui vale ressaltar que apesar das limitações, o estudo tem méritos. É difícil realizar um estudo randomizado, controlado dentro de um cenário desses, que envolve coletas fora de um laboratório específico e com adversidades climáticas. Diante disso, esse é um dos maiores méritos do estudo, fazer um trabalho complexo, com medidas bioquímicas e ecocardiográficas específicas, fora de um ambiente laboratorial. Além disso, acreditamos que os trabalhos desenvolvidos dentro do ambiente prático, atende muito mais as demandas e responde de maneira muito mais completa sobre o comportamento de um fenômeno. É muito importante saber quais são as respostas em relação aos biomarcadores cardíacos e função diastólica da caminhada de longa distância, visto que tal modalidade é uma das que mais cresce no Brasil e no mundo.

Por fim, a atual contribuição de Euzébio et al.^7^ publicado nesta edição dos Arquivos Brasileiros de Cardiologia, além de prover resultados importantes para a literatura científica, também servirá para nortear novos estudos que poderão aperfeiçoar métodos relacionados ao delineamento, coleta de dados e variáveis a serem estudadas. A mensagem final do presente estudo é que indivíduos adultos treinados que participam de eventos de caminhada de longa distância com mais de 240 km de percurso total, não sofrem prejuízo cardiovascular, analisados por meio de biomarcadores cardíacos e variáveis ecocardiográficas.
