# Supplementary material for: Caminhada de Longa Duração pode Alterar Biomarcadores Cardíacos e Variáveis Ecocardiográficas Relacionadas à Função Diastólica?
Source: Arq Bras Cardiol. 2020 Oct 13;115(4):628–9. [Article in Portuguese] doi: 10.36660/abc.20200457 (PMC8386973; doi:10.36660/abc.20200457)
Supplement: Translation

# Translation: Can Long-Distance Walking alter Cardiac Biomarkers and Echocardiographic Variables Related to Diastolic Function?

**Affiliations:** 1 Porto AlegreRS Brazil Programa de Pós-graduação em Ciências da Saúde: Cardiologia e Ciências Cardiovasculares - Universidade Federal do Rio Grande do Sul (UFRGS), Porto Alegre, RS – Brazil

**Keywords:** Walking, Physical Exertion, Biomarkers, Blood Pressure, Troponin T, Natriuretic Peptide Brain, Creatin Kinase, Echocardiography/methods

Walking is one of the most practiced physical activities worldwide. It is also included in physical exercise programs aiming at improving the health-related parameters in different populations.^1^^,^^2^ Therefore, the demand for amateur events encompassing walking has been increasing, especially those related to nature and the culture of a specific region. One of the best known amateur events in the world that involves walking is the “*Camino de Santiago de la Compostela*”. Moreover, here in Brazil, we have the “*Rota das Missões*”, located in the south of the country. The trajectories can have different distances, reaching nearly 40km of walking on the same day. These are two examples of activities that involve walking and add a cultural experience to the event.

In addition to the two abovementioned events, there are long-distance ecological hikes, where participants experience direct contact with nature, going through different terrain characteristics on the same day, reaching a total walking distance of up to 250 km during a 4-day event. We know that each individual responds differently to a certain stimulus; however, walking more than 50 km in one day going up and down slopes, on rough terrain, can have a negative physiological impact on any individual who undergoes this type of modality.^3^ In addition to the physiological damage, we are also concerned with the impact on the cardiovascular system in relation to the volume and intensity of this type of modality.^4^^,^^5^

Among the best known physiological markers, one can mention alterations in cardiac biomarkers such as Creatine Kinase-MB fraction (CK-MB), cardiac Troponin T (cTnT) and the NT-proBNP (N-terminal pro B-type natriuretic peptide), which may be related to myocardial damage.^4^ Among the markers of altered cardiac function, we can highlight some echocardiographic variables, such as early (E) and late transmitral diastolic velocities (A), as well as the E / A ratio, as well as early diastolic velocity by tissue-Doppler (E'), all of which can be used to analyze diastolic dysfunction.^6^

The available scientific evidence on the effects of long-distance walking on cardiac biomarkers and diastolic function shows divergent results regarding cardiovascular risk for this type of modality. In this issue of the journal, Euzébio et al.^7^ explore this topic in long-distance walking participants (mean age 46 ± 10.5). The authors verified these effects on the diastolic function behavior using the E, A waves and E / A ratio, in addition to the cardiac biomarkers CK-MB, Troponin-T and NT-proBNP. They also assessed correlations between diastolic function variables, cardiac biomarkers and whether there is a correlation between some diastolic function variable and some cardiac biomarker. For this purpose, the authors performed a longitudinal study with 25 adult participants (all men), after four women were excluded for not meeting the previous selection criterion, which consisted in walking 56 km, divided into two days, in up to three hours and ten minutes for men and three hours and 30 minutes for women. The evaluations were divided into five stages: A0, the moment before the event started, A1, the first day after the end of the trajectory; A2, the second day after the end of the trajectory; A3, the third day after the end of the trajectory; and A4, the fourth day after the end of the entire trajectory. The total trajectory comprised a distance of 244.7 km. As the main results, the authors found significant increases in CK-MB from A0 to A2, NT-proBNP from A0 to A1, A2, A3 and A4 and E' wave from A0 to A1. Interestingly, and unlike other studies, Troponin-T did not show any significant differences, as well as E and A waves. Moreover, positive correlations were identified between CK-MB and NT-proBNP, CK-MB and Troponin-T, between E/A ratio and E', and a negative correlation between CK-MB and E/A ratio.

Overall, the present study discloses important results on the effects of long-distance walking on parameters related to cardiac biomarkers and diastolic function, demonstrating that, despite the observed alterations, there are no criteria suggestive of myocardial damage, mainly because they did not find any alterations in Troponin-T, E wave and A wave.^7^^,^^8^ The alterations found in NT-proBNP serum levels demonstrate the natriuretic effect on the physiological mechanism of acute and subacute adaptation of the cardiovascular system in relation to exertion.^9^ It is also noteworthy that the increase in serum levels of CK-MB may be related mainly to higher temperatures during certain parts of the trajectory (that varied between 19 and 32ºC), and also to the type of terrain, distance walked and intensity in each part of the trajectory. Indeed, the variation found in this variable was between A0 and A2, predominantly comprising a downslope, promoting greater eccentric muscle contraction, and directly related to greater muscle damage, which may explain this behavior, in addition to a cumulative effect between the A1 and A2 trajectory.^10^ Regarding the diastolic function, the significant increase only in the E' wave between A0 and A1 can be explained by the studied population. It is known that individuals with less physical conditioning are more susceptible to alterations in diastolic function when exposed to physical effort, when compared to individuals with more physical conditioning. Moreover, regular physical training can minimize acute alterations in diastolic function in relation to greater cardiovascular demands during prolonged and intense exercise.^11^ In summary, one needs to pay attention mainly to the behavior of Troponin-T, which is specific to identify myocardial damage due to ischemia, demonstrating that the damage to the cardiovascular system seems to be minimal and that this type of exercise seems to be safe.^12^

Regarding the observed correlations, we can highlight the negative association between CK-MB and E/A (ratio between the rapid ventricular filling velocity and the atrial contraction velocity). One of the assumptions regarding this association can be attributed to the reversible diastolic dysfunction picture as one of the main mechanisms of CK-MB increase in plasma, with a consequent reduction in E wave and increase in A wave. However, this issue still needs to be better explored regarding the associations between cardiac biomarkers and diastolic function from the perspective of long-distance walking.

The study has some limitations, among which we can highlight the lack of standardization in relation to the time of data collection during the days of the event, since this can directly interfere with the assessments of the acute levels of cardiac biomarkers. It would also be important to collect the same variables at rest always on the day after the trajectory. However, the authors point out that this was not feasible due to the time that the journey started (4:00 am). Here, it is worth mentioning that despite the limitations, the study has its strengths. It is difficult to perform a randomized, controlled study in this scenario, which involves data collections outside a specific laboratory and with climatic adversities. Considering this fact, this is one of the greatest merits of the study, carrying out a complex investigation, with specific biochemical and echocardiographic measurements, outside a laboratory environment. Moreover, we believe that studies developed in the practical environment, further meets the demands and responds much more comprehensively about the behavior of a phenomenon. It is very important to know what the responses are in relation to cardiac biomarkers and diastolic function of long-distance walking, as this modality is one of the fastest growing in Brazil and worldwide.

Finally, the contribution of Euzébio et al.^7^ published in this issue of the Brazilian Archives of Cardiology, in addition to providing important results for the scientific literature, will also be used to guide new studies that may improve the methods related to the design, data collection and variables to be studied. The final message of the present study is that trained adult individuals who participate in long-distance walking events comprising more than 240 km of total distance, do not suffer cardiovascular damage, analyzed through cardiac biomarkers and echocardiographic variables.
